# Apologies in Crisis: The Link Between Perceived Burdensomeness and Suicide Risk in Online Text Communication

**DOI:** 10.1111/sltb.70063

**Published:** 2025-11-07

**Authors:** Kenta Ishikawa, Takato Oyama, Yoshihiko Tanaka, Matia Okubo, Hajime Sueki

**Affiliations:** ^1^ Department of Psychology, Faculty of Liberal Arts Teikyo University Tokyo Japan; ^2^ Faculty of Human Informatics Aichi Shukutoku University Aichi Japan; ^3^ Department of Psychology Senshu University Kanagawa Japan; ^4^ Research Fellow of the Japan Society for the Promotion of Science Tokyo Japan; ^5^ Faculty of Human Sciences Wako University Tokyo Japan

**Keywords:** apology, perceived burdensomeness, suicide assessment, suicide risk, text communication

## Abstract

**Introduction:**

Suicide is a leading cause of preventable death worldwide, with approximately 700,000 individuals dying by suicide annually. Recent studies have highlighted the importance of text‐based risk assessments and the use of artificial intelligence technologies such as ChatGPT in suicide prevention. This study examined how intolerable interpersonal situations influence text communication, focusing on the relationship between perceived burdensomeness and the expression of apologetic messages.

**Methods:**

A total of 120 university students participated in the Interpersonal Persistence Task (IPT), manipulating levels of perceived burdensomeness and thwarted belongingness (high and low PB–TB conditions) to observe their effects on participants' desire to escape and communication behaviors.

**Results:**

Participants in the high PB–TB condition reported higher levels of perceived burdensomeness and a stronger desire to escape the task. Regression analyses indicated that perceived burdensomeness predicted the frequency of apology expressions in the high PB–TB condition.

**Conclusions:**

These findings suggest that perceived burdensomeness may influence the expression of apology in digital communication under interpersonal distress. While not directly indicative of suicide risk, apologetic messages could reflect psychological vulnerability in certain contexts.

## 1 Introduction

Suicide is one of the leading causes of preventable deaths worldwide, affecting approximately 700,000 individuals annually (World Health Organization 2021). Death by suicide is not only tragic but also profoundly affects loved ones left behind. To prevent suicide, suicide risk should be assessed and intervention strategies developed for high‐risk individuals. Previous studies have reported various types of online suicide risk assessments, including search‐linked advertising (Arendt et al. 2020; Sueki 2015; Sueki and Ito 2018), text‐based assessments during email or chat‐based counseling sessions (Coleman et al. 2019; Galfalvy and Gould 2022; Gould et al. 2021; Sueki et al. 2023), and smartphone screen analysis using Optical Character Reader (OCR) technology to extract textual information for estimating suicide risk (Ammerman et al. 2025). Furthermore, recent years have witnessed significant advancements in the use of AI technologies such as ChatGPT in consultative roles, as highlighted in studies by Arendt et al. (2023) and Bernert et al. (2020), which have demonstrated the growing importance of developing text‐based online risk assessments and intervention strategies. These approaches have distinct advantages such as scalability, automation, and access to naturalistic language. However, they also have limitations in terms of context sensitivity, interpretability, and clinical validation. To address these limitations, it is essential to identify specific linguistic content that reliably indicates suicide risk.

Conducting text‐based counseling services necessitates assessing the content of the text to estimate the suicide risk among clients (Galfalvy and Gould 2022; Gould et al. 2021; Sueki et al. 2023). For example, Sueki et al. (2023) conducted a suicide risk assessment based on the characteristics of messages exchanged as part of Internet counseling services, finding that messages from clients with high suicidal ideation tended to include references to suicide, feelings of being a burden to others, and self‐loathing (Sueki et al. 2023). These results align with the interpersonal theory of suicide, which hypothesizes that a decreased sense of belonging and increased perceived burdensomeness can trigger suicide‐related behaviors (Joiner 2005; Van Orden et al. 2008). According to this theory, suicidal desire arises when individuals simultaneously experience a sense of not belonging and the belief that they are a burden to others (Joiner 2005; Van Orden et al. 2008).

To investigate these interpersonal factors under controlled conditions, Collins et al. (2016) developed the Interpersonal Persistence Task (IPT), which ethically simulates social adversity by manipulating participants' perceived burdensomeness and thwarted belongingness. From the perspective that suicide offers an escape from intolerable pain (Baumeister 1990; O'Connor 2011), Collins et al. (2016) manipulated intolerable situations (by increasing perceived burdensomeness and thwarted belongingness) and measured the desire to escape (quit) using an Interpersonal Persistence Task (IPT). In this task, participants were required to achieve a target score for word identification through message exchanges within a team of three members. However, only one participant underwent the experiment; the scores and interactions of other team members were computer‐based programs. In intolerable conditions, participants' performance was manipulated to be lower than their actual performance, and they received critical and blaming messages from other team members. These manipulations increased the levels of thwarted belongingness and perceived burdensomeness among participants. The results revealed a positive correlation between the desire to escape and the likelihood of suicide (Collins et al. 2016). Subsequent studies using the IPT have confirmed the validity of experimental manipulation, suggesting that a diminished sense of belonging and perceived burdensomeness increase the desire to escape (Collins et al. 2016, 2017; Kyron et al. 2019). However, these studies did not analyze the actual linguistic content of the participants' written messages (Collins et al. 2016, 2017; Kyron et al. 2019). As a result, it remains unclear how heightened burdensomeness and thwarted belongingness influence the expression of apology or other text‐based markers of distress. This gap is particularly important in online counseling contexts, where practitioners must often infer suicide risk from written messages alone. By focusing on how such states shape language use, this study seeks to bridge the gap between internal psychological risk and observable communication patterns that may signal vulnerability to suicide.

This study aims to investigate the characteristics of text communication under intolerable situations using the IPT, focusing on the relationship between messages of “apology” and suicide risk, as previous studies have revealed that apologetic messages are a common theme in suicide notes (Foster 2003; Foster et al. 1997; Izawa 2002; Mejías‐Martín et al. 2023; Namratha et al. 2015). Suicide notes are documents left by an individual who intends to die or has died by suicide, comprising important information about intentions and reasons for suicide. Foster (2003) investigated the themes of 42 suicide notes from a psychological autopsy study, revealing that apology/shame was the most common theme. A high proportion of apologetic expressions in suicide notes has also been reported in other cultural contexts (Izawa 2002; Mejías‐Martín et al. 2023; Namratha et al. 2015). Apologies in suicide notes represent increased perceived burdensomeness to others and family members (Foster 2003; Foster et al. 1997; Mejías‐Martín et al. 2023; Namratha et al. 2015). Furthermore, Syme and Hagen (2019) conducted a cross‐cultural analysis of 473 ethnographic records from 53 cultures, showing that some suicidal acts may serve as a costly signal of apology—that is, a communicative act expressing guilt and an attempt to repair social relationships. This interpretation highlights that suicidal behavior can function not only as an escape from pain but also as a form of apology, thereby complementing the interpersonal theory of suicide, which posits that perceived burdensomeness can trigger suicidal behaviors (Joiner 2005; Van Orden et al. 2008).

Based on previous studies (Collins et al. 2016, 2017; Foster 2003; Foster et al. 1997; Izawa 2002; Mejías‐Martín et al. 2023; Namratha et al. 2015), we examine the effect of perceived burdensomeness and thwarted belongingness on text communication in intolerable situations, hypothesizing that intolerable situations tend to increase individuals' perceived burdensomeness and diminish belongingness, leading to feelings of apology toward others. To test this hypothesis, we used the IPT to manipulate the levels of perceived burdensomeness and thwarted belongingness (through high and low perceived burdensomeness–thwarted belongingness [PB‐TB] conditions) among participants (Collins et al. 2016). Additionally, as part of a broader research project, participants completed self‐report measures assessing anxiety, depression, and social evaluative concerns. While these data are not analyzed in the present study, they were included to support future investigations of individual differences in psychological vulnerability. We predicted that participants would express more apologies toward other team members when their perceived burdensomeness and thwarted belongingness were increased (i.e., high PB‐TB condition) than when they were decreased (i.e., low PB‐TB condition).

## 2 Method

### 2.1 Participants

We recruited 120 university students (70 women and 50 men, M_age_ = 19.08 years, SD = 1.19) for this experiment. Participants were recruited from multiple departments (e.g., humanities, education, or economics) at the same university. The study was announced during regular class sessions. Participation was voluntary and offered in exchange for partial course credit. Before the study, we conducted a priori power analysis to determine the required sample size using G*Power 3.1 (Faul et al. 2007), assuming a significance level of α = 0.05 and an effect size of *r* = 0.3, referencing Cohen's (1988) criteria. The required sample size was determined to be 84 participants. Considering a potential 30% dropout rate (Collins et al. 2016), we recruited 120 participants. This study was approved by the Research Ethics Review Board in the Graduate School of Psychology at Wako University (No. 2021_004). The experiments were conducted in accordance with the principles of the Declaration of Helsinki. We obtained written informed consent from all participants.

### 2.2 Questionnaires

We used several questionnaires (e.g., State–Trait Anxiety Inventory, PHQ9, and the Beck Depression Inventory II) to assess psychological disorders associated with suicide. The analysis of these questionnaires is reserved for a separate study.

### 2.3 Procedure

We used the IPT—a three‐player computer task—originally developed by Collins et al. (2016). It is designed to not only manipulate the levels of participants' PB‐TB but also their desire to escape from the task by controlling task performance and feedback from other team members (Collins et al. 2016).

Participants were seated in a dimly lit room approximately 57 cm away from the screen and instructed to perform the task as a team of three online players. They were asked to judge whether two blue letters (e.g., Y and U) presented on the screen were identical by pressing keys as quickly and accurately as possible. Participants were instructed that they would gain and lose one point for each correct and incorrect response, respectively. Their goal was to earn a score higher than the target score, which was the average score of previous participating teams. The word‐discrimination task consisted of six rounds, each containing 15 trials. Therefore, the total number of trials was 90.

**FIGURE 1:**
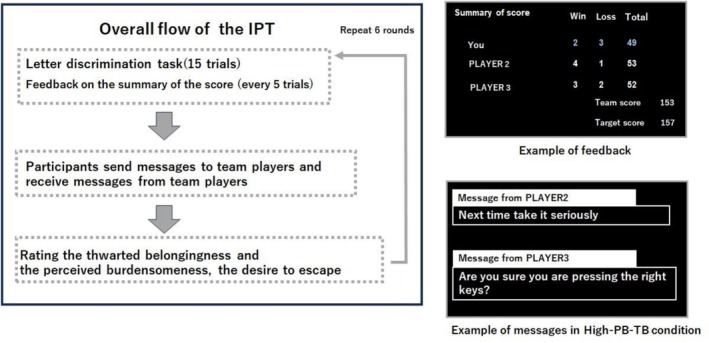
The Overall flow of the IPT task and examples of the presentation of feedback and messages.

The IPT manipulated participants' sense of perceived burdensomeness by presenting the score table at the end of each round (see Figure 1), displaying the scores of participants and their team members, the total score of the teams, and the target score.

Participants were randomly assigned to either the high or low PB–TB condition (60 participants per group) using a computerized randomization scheme. In the high PB‐TB condition, the participants' scores were manipulated to be significantly lower than those of their team members (i.e., 40% success rate), while those scores were manipulated to be equal to or better (i.e., 60% success rate) than their team members in the low PB‐TB condition. Therefore, participants in the high PB‐TB condition were led to believe that they did not contribute to their team, making them feel like a burden to their team members. The IPT manipulated the sense of belonging through feedback messages from each team member. At the end of each round, participants were asked to send a short message to each team member (i.e., players 2 and 3), as this feedback could help improve their team's performance. After participants sent messages to their team members, they received pre‐typed replies from them (Figure 1). In the high PB‐TB condition, these messages were increasingly critical and blaming, whereas in the low PB‐TB condition, the messages were supportive and encouraging. Thus, participants in the high PB‐TB condition may have felt undervalued by their team members, leading them to believe that they did not belong in the team.

At the end of each round, participants were asked to rate their sense of belonging, perceived burdensomeness toward their team members, and desire to escape the task on a seven‐point scale ranging from “0 = not at all” to “6 = strongly”. At the end of the experiment, we identified suspicion by explicitly asking participants if they believed they were playing as part of a computer‐generated team.

### 2.4 Coding of Apologetic Messages

According to Izawa's (2002) criteria, we classified Japanese phrases such as “ごめん”, “ごめんなさい”, “申し訳ない”, “申し訳ないです”, and “すいません” as expressions of apology in the present study. These phrases are commonly translated into English as variations of “sorry” or “apology”. Two graduate students in a clinical psychology program coded whether each participant's messages to team members (i.e., players 2 and 3) contained expressions of apology (presence = 1, absence = 0). The two coders were unaware of the purpose and hypothesis of the study. As each participant sent messages across the six blocks to the remaining two team members, the total number of messages was 12 per participant. The judgments of the two coders were consistent across all apologetic messages.

### 2.5 Statistical Analysis

All statistical analyses were conducted using JASP version 0.19.3.0 (JASP Team 2024). To assess whether perceived burdensomeness and thwarted belongingness affected the desire to escape, we performed a mixed two‐factor ANOVA with condition (high vs. low PB‐TB) as a between‐subjects factor and block (Blocks 1–6) as a within‐subjects factor. Additionally, internal consistency was excellent for all three scales: Cronbach's alpha was 0.95 for thwarted belongingness, 0.95 for perceived burdensomeness, and 0.97 for the desire to escape.

To analyze apologetic expressions, we first compared the mean apology rates between groups using an independent‐samples t‐test. Then, for each condition, we conducted a multiple regression analysis to examine whether participants' perceived burdensomeness and thwarted belongingness scores predicted the frequency of apology messages.

## 3 Results

One participant was excluded from the analysis due to equipment malfunctions. In addition, 38 participants were excluded because they were aware of the experimental manipulation—18 from the high PB‐TB condition (31%) and 20 from the low PB‐TB condition (33%). These proportions were comparable across conditions, suggesting that the exclusions did not introduce systematic bias. Therefore, the final sample included 81 participants (49 females, 32 males; *M*_age_ = 19.06 years, SD = 1.06), with 41 assigned to the high PB‐TB condition and 40 to the low PB‐TB condition. Age and gender distributions did not significantly differ between the two groups (Age: *t*(79) = 1.57, *p* = 0.12, *d* = 0.35; Sex: χ^2^(1) = 2.25, *p* = 0.13).

To assess whether the results of Collins et al. (2016) could be replicated, we analyzed the effects of perceived burdensomeness and thwarted belongingness on the desire to escape by perfectly replicating the results of previous studies (Collins et al. 2016, 2017; Kyron et al. 2019), indicating that the desire to escape scores increased linearly in the high PB‐TB condition. A detailed analysis of the rating scores of thwarted belongingness, perceived burdensomeness, and desire across Blocks 1–6 is reported in the Supplemental Data (see **Supplement A**).

### 3.1 Analysis of Participants' Messages to Team Members

We calculated the means of the presence of apology messages in all blocks and conducted an independent‐sample *t*‐test to compare their occurrence between the high and low PB‐TB conditions. The results revealed that the mean rate of apology messages was higher in the high‐PB‐TB condition (*M* = 34.35%, SD = 27.27) than in the low PB‐TB condition (*M* = 1.88%, SD = 5.16), *t*(79) = 7.40, *p* < 0.001, Cohen's *d* = 1.65. To further clarify the relationships between the rates of apologetic messages and interpersonal risk factors, we also conducted correlational analyses. The results showed that the rates of apologetic messages were strongly and negatively correlated with thwarted belongingness (*r* = −0.56, *p* < 0.001), and positively correlated with perceived burdensomeness (*r* = 0.69, *p* < 0.001).

To assess the effects of thwarted belongingness and perceived burdensomeness on the presence of apology messages, we conducted separate multiple regression analyses for the high and low PB‐TB conditions, in which thwarted belongingness and perceived burdensomeness served as independent variables and the average rate of apology messages was the dependent variable.

**FIGURE 2:**
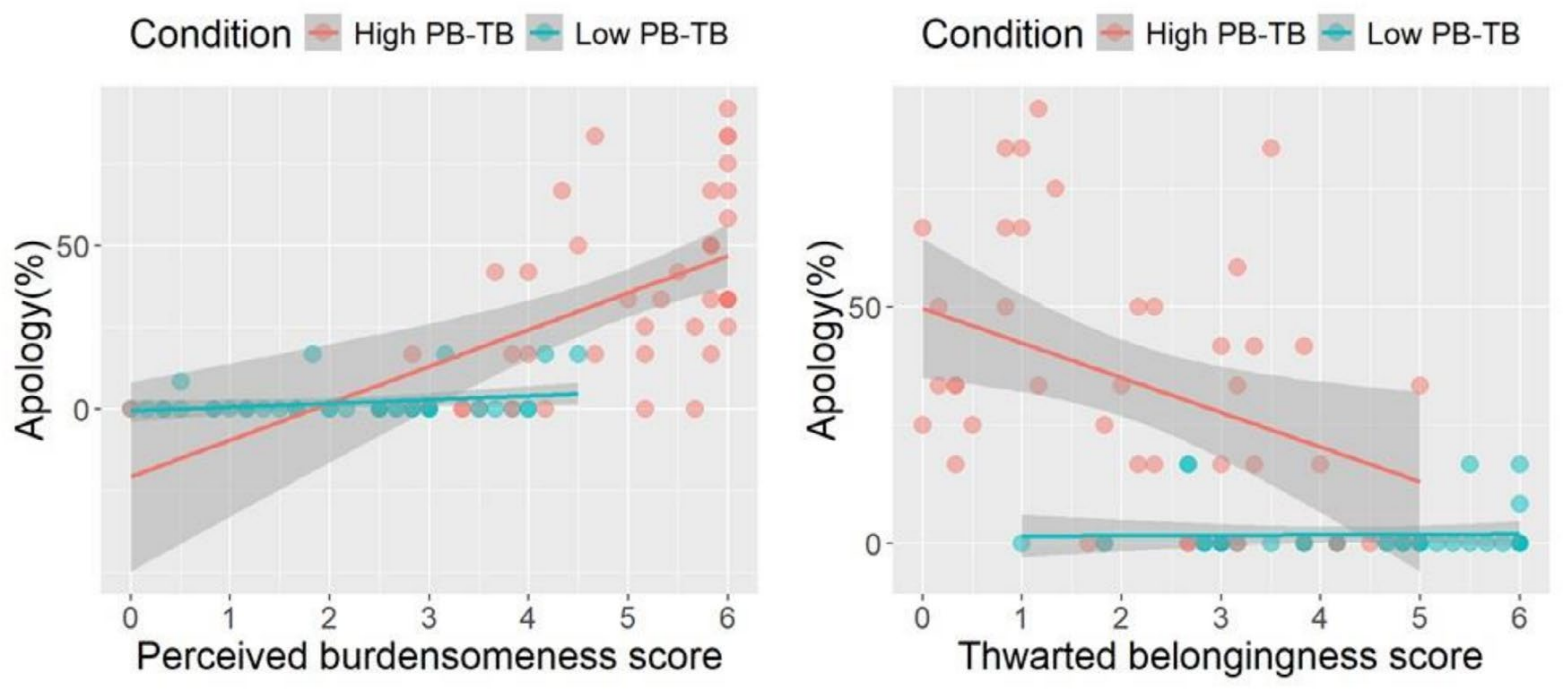
Scatter plot representing the relationship rates of the apology message, score of perceived burdensomeness (left), and score of thwarted belongingness (right). *Note:* Red and green dots indicate participants in the high and low PB‐TB conditions, respectively. The gray band represents the 95% confidence interval.

Importantly, the model was statistically significant in the high PB‐TB condition (*R* ^2^ = 0.278, *F* (2,38) = 7.314, *p* = 0.002). As Figure 2 reveals, the increased perceived burdensomeness score significantly predicted the presence of the apology message (*β* = 0.461, *t* = 3.06, *p* = 0.004). No significant relationship was observed between thwarted belongingness and the presence of apologetic messages (*β* = −0.13, *t* = −0.864, *p* = 0.393). In the low‐PB‐TB condition, the model did not significantly explain the variance in the presence of apologetic messages, *R* ^2^ = 0.077, *F* (2,37) = 1.549, *p* = 0.23 (perceived burdensomeness: *β* = 0.28, *t* = 1.76, *p* = 0.088; thwarted belongingness: *β* = 0.05, *t* = 0.315, *p* = 0.75).

### 3.2 Exploratory Analysis of Group × Predictor Interactions

We calculated the means of the presence of apology messages in all blocks. To further examine whether the predictive relationships differed between groups, we conducted an exploratory regression analysis including group × predictor interaction terms. In this model, the group variable was coded as a dummy variable (high PB–TB = 1, low PB–TB = 0), and the predictors (perceived burdensomeness and thwarted belongingness) were mean‐centered. The overall model was significant, *R* ^2^ = 0.59, *F* (5, 75) = 21.87, *p* < 0.001. Importantly, the interaction between perceived burdensomeness and group was significant (*β* = 8.51, SE = 3.14, *p* = 0.008), indicating that the association between perceived burdensomeness and apology frequency was stronger in the high PB–TB group. By contrast, the interaction between thwarted belongingness and group was not significant (*β* = −3.86, SE = 2.88, *p* = 0.185).

## 4 Discussion

This study examined the effects of thwarted belongingness and perceived burdensomeness on text communication using an interpersonal persistence task (IPT). The results revealed that an increased perceived burdensomeness score was significantly associated with the presence of an apology message on the IPT, supporting our prediction that messages from participants to team members in the high‐PB‐TB condition exhibited a high rate of apology messages. Sueki et al. (2023) reported that texts from clients with high suicidal ideation tend to include references to suicide and perceived burdensomeness to others. Previous studies also reported that apologies were one of the typical and most important themes in suicide notes (Foster 2003; Foster et al. 1997; Mejías‐Martín et al. 2023; Namratha et al. 2015). Furthermore, apologies are associated with feelings of shame and self‐loathing, which are emotions strongly experienced by individuals at high risk of suicide (Foster 2003; Sueki et al. 2023). In intolerable situations, an increase in expressions of apology is related to heightened perceived burdensomeness to others, which may reflect heightened interpersonal distress and could serve as potential markers of vulnerability.

The results also revealed that the thwarted belongingness score was not associated with the rates of apologies. Apologies are emotional responses that occur when individuals recognize their faults and the inconvenience they have caused others. Therefore, it makes sense that thwarted belongingness did not strongly influence the expression of apologies. In contrast, perceived burdensomeness was associated with an increase in expressions of apology. These findings suggest that perceived burdensomeness may be more closely associated with apologetic behavior than thwarted belongingness. This interpretation is consistent with previous findings showing that perceived burdensomeness is a stronger mediator of the desire to escape (Kyron et al. 2019) and is more strongly linked to suicidal ideation than other interpersonal risk factors (Ma et al. 2016). These findings support the relevance of perceived burdensomeness as a psychological construct in understanding responses to interpersonal distress. This study is the first to experimentally demonstrate that perceived burdensomeness can elicit apologetic expressions under simulated interpersonal adversity, providing an empirically grounded link between psychological risk states and observable linguistic behavior.

The present study has several theoretical and clinical implications related to suicide. First, the expression of apologies may be elicited by heightened perceived burdensomeness in intolerable situations. In such contexts, apologies may reflect internalized self‐blame or efforts to repair perceived interpersonal harm, which are psychological responses commonly observed in individuals experiencing suicidality. This interpretation is consistent with previous studies reporting that apologies frequently appear in suicide notes (Foster 2003; Foster et al. 1997; Mejías‐Martín et al. 2023; Namratha et al. 2015). Previous studies primarily used the Interpersonal Persistence Task (IPT) to examine psychological risk factors for suicide (Collins et al. 2016, 2017; Kyron et al. 2019). Building on this work, the present study extends the utility of the IPT by incorporating the analysis of linguistic behavior under induced interpersonal distress. Specifically, we focused on expressions of apology as observable markers of psychological strain. In digital suicide prevention settings—such as chat‐ or email‐based counseling—assessing acute risk in real time remains a critical challenge. Our findings help bridge the gap between internal psychological states and external behavioral cues by identifying language features that may signal distress in ethically simulated high‐risk scenarios. These findings contribute to the refinement of interpersonal theories of suicide by linking perceived burdensomeness to specific linguistic behaviors in controlled settings. Furthermore, with the advancement of AI‐based language processing and passive sensing technologies (Ammerman et al. 2025), such indicators may eventually support automated risk detection systems for suicide prevention in digital environments.

This study has several limitations. First, the sample consisted solely of Japanese university students, limiting generalizability to other cultural or high‐risk populations. In Japanese culture, apologies often function as social conventions rather than admissions of personal fault (Heine 2014; Maddux et al. 2011), which may influence their association with perceived burdensomeness. Cross‐cultural research, however, suggests that some suicidal behaviors serve interpersonal functions such as sincere apology (Syme and Hagen 2019; Takahashi 1997). This indicates that the link between apology and suicide is not culturally unique. Even so, it remains uncertain whether apologies in experimentally induced conditions with limited interpersonal consequences reflect the same psychological dynamics as those found in suicidal states. Apologizing can stem from politeness or perceived criticism rather than psychological distress. For example, in Japan, it is common to apologize even for minor social disruptions, such as interrupting a conversation or being slightly late, without experiencing significant guilt or emotional strain. This normative use of apology as a social script or politeness strategy may obscure its diagnostic value as an indicator of psychological vulnerability, particularly in cross‐cultural research. Thus, further study is needed to clarify which forms of apologetic expression, particularly in combination with other cues, are linked to psychological vulnerability and suicide risk. Second, the study involved low‐intimacy interactions, unlike real‐world contexts such as suicide notes or counseling, where deeper interpersonal meaning is involved. Although the IPT provides a valid simulation of interpersonal distress (Collins et al. 2016, 2017; Kyron et al. 2019), it may not fully reflect the chronic experiences of individuals at high suicide risk. Furthermore, this study relied on brief text exchanges, which may not fully capture the dynamics of real‐world communication, such as those in online counseling. In addition, although depressive symptoms and suicidal ideation were assessed using standardized questionnaires (BDI‐II, PHQ‐9), only a very small number of participants endorsed suicidal thoughts. This low endorsement limits the generalizability of our findings, as the observed increase in apologetic expressions may not directly translate to individuals experiencing clinically significant suicidality. Future research should examine both more naturalistic, interactive communication settings and high‐risk populations to clarify whether similar linguistic patterns emerge. Third, the apologies expressed in this study may reflect common social conventions or responses to perceived criticism, rather than perceived burdensomeness. Future research should identify which types of apologies are indicative of elevated suicide risk. Additionally, while the present study focused solely on apologetic expressions elicited in an experimental context, other linguistic features may also reveal signs of psychological vulnerability or resilience under distress. Finally, although our a priori power analysis indicated that 84 participants would be required, the final sample size was 81. This shortfall means that the study did not fully reach the predefined target, which should be considered a limitation. However, the observed correlations with apology expression were substantial (thwarted belongingness: *r* = −0.56; perceived burdensomeness: *r* = 0.69), exceeding the medium effect size assumed in the power analysis (*r* = 0.30). This suggests that the statistical power of the study was sufficient despite the slightly smaller sample size.

## 5 Conclusion

This study is the first to experimentally manipulate a state close to the immediate pre‐suicide condition and detect it not only as a psychological state (i.e., perceived burdensomeness and thwarted belongingness) but also as a specific communicative behavior involving sending apologetic messages. The IPT could serve as a foundation to experimentally evaluate effective crisis interventions in the future. When aiming to automate crisis interventions using artificial intelligence, it is crucial to experimentally investigate the types of communication that increase or decrease suicide risk. Our findings play an important role in expanding the models of suicide by uncovering the relationships between communicative behavior and the psychological state of individuals in intolerable situations. These results suggest that sending an apology message can be an important sign that a person is experiencing a crisis. Although further research is needed to validate such signs across diverse contexts and populations, our findings highlight the importance of integrating interpersonal risk indicators into emerging tools for automated suicide risk assessment.

## Author Contributions

**Kenta Ishikawa:** conceptualization (lead), data curation (lead), formal analysis (lead), funding acquisition (lead), investigation (equal), methodology (equal), resources (lead), validation (lead), visualization (equal), writing – original draft (lead), writing – review and editing (lead). **Takato Oyama:** conceptualization (supporting), data curation (equal), formal analysis (equal), methodology (supporting), writing – original draft (supporting), writing – review and editing (supporting). **Yoshihiko Tanaka:** data curation (supporting), formal analysis (supporting), investigation (supporting), writing – original draft (supporting), writing – review and editing (supporting). **Matia Okubo:** conceptualization (supporting), supervision (supporting), writing – original draft (supporting), writing – review and editing (supporting). **Hajime Sueki:** conceptualization (supporting), methodology (supporting), supervision (supporting), writing – original draft (supporting), writing – review and editing (supporting).

## Ethics Statement

This study was approved by the Research Ethics Review Board in the Graduate School of Psychology at Wako University (No. 2021_004). The experiment was conducted in accordance with the principles of the Declaration of Helsinki. Written informed consent was obtained from all participants.

## Conflicts of Interest

The authors declare no conflicts of interest.

## Supporting information


**Appendix S1:** Supporting Information

## Data Availability

Behavioral and supplemental data are available at the following link: https://osf.io/tgk6v/.
