# Supplementary material for: Apologies in Crisis: The Link Between Perceived Burdensomeness and Suicide Risk in Online Text Communication
Source: Suicide Life Threat Behav. 2025 Nov 7;55(6):e70063. doi: 10.1111/sltb.70063 (PMC12593273; doi:10.1111/sltb.70063)
Supplement: Supplementary file 1 — Appendix S1: Supporting Information [file SLTB-55-0-s001.docx]

*Thwarted Belongingness*

We calculated mean scores of thwarted belongingness across all blocks and conducted a mixed two-factor Analysis of Variance (ANOVA). The analysis used the experimental condition (high and low-PB-TB condition) as a between-subjects factor and Block (blocks 1–6) as a within-subjects factor. The ANOVA revealed a significant main effect of the condition, *F* (1, 79) = 53.64, *p* < .001, η^2^_p_ = .40, indicating that mean scores of belongings were lower for the high-PB-TB condition (*M* = 2.09, *SD* = 1.39) than for the low-PB-TB condition (*M* = 4.33, *SD* = 1.36). There was a significant main effect of the block (*F* (5, 395) = 4.554, *p* < .001, η^2^_p_ = .06). Importantly, we found a two-way interaction between the condition and the block (*F* (5, 395) = 10.97, *p* < .001, η^2^_p_ = .12). To further clarify the two-way interaction, we performed a separate one-factor ANOVA for each condition with Block as the within-subjects factor. As Figure 1 shows, significant main effects of Block were observed along with a linear trend in both the high-PB-TB condition (*F* (5, 200) = 9.23, *p* < .001, η^2^_p_ = .19) and the low-PB-TB condition (*F* (5, 195) = 5.36, *p* < .001, η^2^_p_ = .12).

Figure 1. The result of the self-reported ratings in the high PB-TB condition and the low PB-TB condition across blocks 1 to 6.


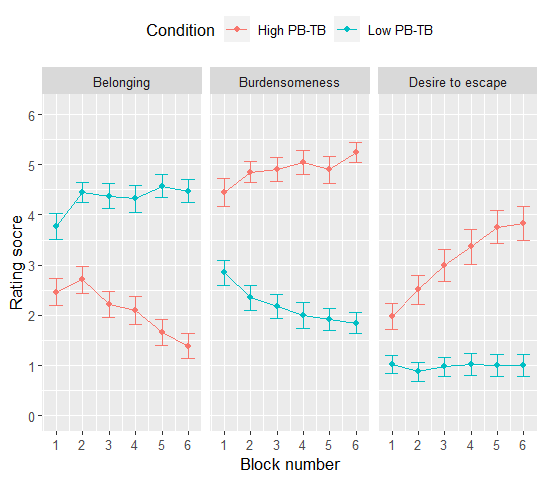


*Perceived burdensomeness*

Mean scores of the perceived burdensomeness were calculated across all blocks and subjected to the mixed two-factor ANOVA with the condition as a between-subjects variable and Block as a within-subjects factor. The results showed that the main effect of the condition was significant, *F* (1, 79) = 92.54, *p* < .001, η^2^_p_ = .54, indicating that mean scores of perceived burdensomeness were higher for the high-PB-TB condition (*M* = 4.90, *SD* = 1.30) than for the low-PB-TB condition (*M* = 2.19, *SD* = 1.22). There was no significant main effect of the block (*F* (5, 395) = 0.56, *p* = .73, η^2^_p_ = .007). We found the two-way interaction between the condition and the block, *F* (5, 395) = 8.93, *p* < .001, η^2^_p_ = .10. To further clarify this interaction, we performed the separate one-factor ANOVA for each condition with Block as the within-subjects factor. As Figure 1 shows, significant main effects of Block were observed along with a linear trend in both the high-PB-TB condition (*F* (5, 200) = 3.34, *p* = .006, η^2^_p_ = .08) and the low-PB-TB condition (*F* (5, 195) = 6.07, *p* < .001, η^2^_p_ = .14).

*Desire to escape*

Mean scores of the desire to escape were calculated across all blocks and subjected to the mixed two-factor ANOVA with the condition as a between-subjects variable and the Block as a within-subjects factor. The main effect of the condition was significant *F* (1, 79) = 36.99, *p* < .001, η^2^_p_ = .32, indicating that mean scores of desire to escape were higher for the high-PB-TB condition (*M* = 3.07, *SD* = 1.81) than for the low-PB-TB condition (*M* = 0.98, *SD* = 1.22). There was a significant main effect of the block (*F* (5, 395) = 19.05, *p* < .001, η^2^_p_ = .19). We found the two-way interaction between the condition and the block, (*F* (5, 395) = 17.62, *p* < .001, η^2^_p_ = .18). To clarify this interaction, we conducted the separate one-factor ANOVA for each condition with Block as the within-subjects factor. As Figure 2 shows, significant main effects of Block were observed along with a linear trend in the high-PB-TB condition (*F* (5, 200) = 22.53, *p* < .001, η^2^_p_ = .36). There was no significant main effect of the Block in the low-PB-TB condition (*F* (5, 195) = 0.62, *p* = .68, η^2^_p_ = .02).

Additionally, we have reported internal consistency coefficients for the score of perceived burdensomeness (α = .95), thwarted belongingness (α = .95), and desire to escape (α = .97), which demonstrate excellent reliability across scales in the present study.

To assess whether perceived burdensomeness or thwarted belongingness are strongly associated with the desire to escape, we conducted a forced-entry multiple regression analysis with perceived burdensomeness and thwarted belongingness scores as predictors of the desire to escape. The result showed that a model significantly explained the variance of the desire to escape (*R*² = .525, *F* (2,78) = 43.17, *p* < .001), indicating that the score of perceived burdensomeness (*β* = 0.472, *t* = 4.81, *p* < .001) and thwarted belongingness significantly predicted the desire to escape (*β* = − 0.336, *t* = − 3.43, *p* < .001).

We perfectly replicated the results of the previous studies (Collins et al., 2016, 2017; Kyron et al., 2019), indicating that the score of the desire to escape displayed a linear increase in the high PB-TB condition. This result supports the interpersonal theory of suicide, which suggests that suicidal behaviors can be caused by increased thwarted belongingness and perceived burdensomeness in high-risk groups for suicide (Joiner, 2005; Van Orden et al., 2008). Furthermore, the result of the regression analysis indicated that the desire to escape was strongly associated with perceived burdensomeness than with thwarted belongingness. This result is consistent with the previous study, suggesting that perceived burdensomeness can be a stronger driver of the desire to escape than thwarted belongingness (Kyron et al., 2019; Ma et al., 2016).
